# Avoiding pitfalls in determining antimicrobial activity of plant extracts and publishing the results

**DOI:** 10.1186/s12906-019-2519-3

**Published:** 2019-05-22

**Authors:** Jacobus Nicolaas Eloff

**Affiliations:** Department of Agriculture and Animal Health,College of Agriculture and Environmental Sciences, University of South Africa, Johannesburg, South Africa

**Keywords:** Plant extracts, Agar diffusion assay, Serial microplate dilution. *p*-iodonitrotetrazolium violet, Extractant

## Abstract

**Background:**

There is an urgent need to discover new antimicrobial compounds or extracts to address the crucial problem of increasing microbial resistance against current antibiotics. Plant chemical biodiversity is a valuable potential resource. Although compounds from plants are used as basis for several human drugs, no commercially successful antibiotic has yet been discovered from plants, despite more than a thousand publications in this field per year. This may be due to wrong methods that have been used or wrong plants that were investigated. A lot of energy is wasted by using techniques such as agar diffusion that do not work well with plant extracts. Many manuscripts are rejected before sending to reviewers because wrong methods are used. Antimicrobial activity of plant extracts based on agar diffusion studies have limited value.

**Methods:**

Results obtained from several hundred of our publications in this area as researcher and experience as editor was used to identify difficulties in generating reproducible data. Other publications were also consulted and procedures used were evaluated.

**Results:**

Because many of the antimicrobial compounds in plant extracts are relatively non-polar, these compounds do not diffuse well in the aqueous agar matrix used in agar diffusion studies. So many other factors also influence the zone of inhibition, that results between different laboratories are not comparable. The different methods used to determine the minimal inhibitory concentration (MIC) in serial dilution studies have been discussed. Using *p*-iodonitrotetrazolium violet to indicate growth provided the best results. Factors such as inoculum size, solvent, selection of positive controls and selection of plants to investigate also play a role. A method developed to determine antibacterial and antifungal activity of plant extracts work very well and is widely used based on > 1830 citations.

**Conclusions:**

By using proposed methods manuscripts will provide reproducible information that may be published in good journals. The publications could contribute to a rational basis for finding compounds or extracts from plants that may address the problem of antimicrobial resistance. Random screening of a large number of plant species using this technique have already led to some commercial applications and identification of a potentially new antifungal framework compound.

## Background

The problems encountered with antibiotics are well-formulated by Walsh [1]): ”Every antibiotic that is introduced into clinical use has a limited shelf life as it selects for bacteria that have some intrinsic or acquired mechanism of resistance. Although these bacteria are rare (for example, 1 in 10^8^), in the continuing presence of the selecting antibiotic the resistant bacteria become more populous than their dying neighbours.” The situation is aggravated by non-compliance of patients not completing the treatment dosage or by the indiscriminate use of antibiotic feed additives in food production.

The chief medical officer in Britain stated that the problem of antimicrobial resistance is a greater threat to humanity than global warning [2]. Some authors have warned that we may be approaching the post antibiotic era [3]. Before the discovery of antibiotics, infections were the main cause of deaths in humans. Burki [4] made the following statement: “on current trends—antimicrobial resistance (AMR) would kill 10 million people worldwide each year by 2050 and would cost the global economy US$100 trillion between 2015 and 2050”.

In an excellent review paper Walsh [1] stated that there was golden age of discovery with new classes of antibiotics from natural products discovered in 1935, 1940, 1949, 1950, 1952, 1958, 1962. There was an innovation gap between the introduction of quinolones in 1962 and the approval of oxazolidinone linezolid in 2000. The mining of natural products for new scaffold molecules was very successful, but these were mainly from microorganisms and marine organisms.

**Fig. 1:**
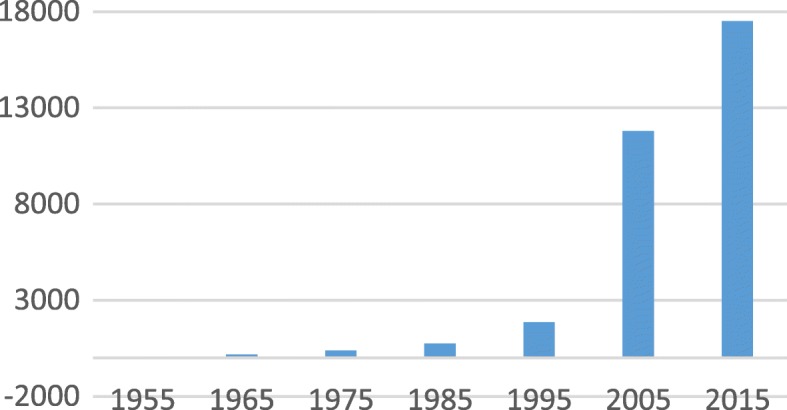
Number of papers listed by Google scholar for 10 year periods since 1945 using “plant AND antimicrobial OR antibiotic OR antibacterial OR antifungal” as search terms

No commercially successful antibiotic has yet been discovered from plants despite the high success rate from plant products in treating human ailments [5, 6] such as cancer, malaria and diabetes. Some food supplements based on plant extracts do have useful antimicrobial activity and have been commercialized, but the aim is not to yield new antibiotics. The aim of most papers investigating the antimicrobial activity of plant extracts is to find new antibiotics that would address the growing resistance against antibiotics. In the order of 25% of medicines prescribed in the USA is based on compounds isolated from plants [4]. The lack of success in finding new antibiotics from plants is not because there was no work on determining the antimicrobial activity of plant extracts [7, 8]. Many papers have been published in journals not accredited by ISI. The number of papers indexed by Google scholar was determining using the keywords, plant AND antimicrobial OR antibiotic OR antibacterial OR antifungal. The number of papers listed for different decades’ changes from 75 in 1945 to 1955 to 17,500 in 2005–2015 (Fig. 1). This large increase indicates the interest in this field, but the growth may be skewed by the large number of very poor journals that have appeared since the development of on-line publications.

One can speculate on why there is such a large difference in success rate in finding commercially useful antimicrobial compounds from plants compared to compounds active against other human and animal diseases since plant are prone to microbial attack but not to many human or animal diseases. A possible reason is that scientists have used the wrong methods or have investigated the wrong plants.

As a section Editor of BMC Complementary and Alternative Medicine and member of Editorial Boards of other journals, I am dismayed by the energy and time wasted by so many authors that do not use acceptable techniques to examine the antimicrobial activity of plant extracts. This leads to a rejection of manuscripts even before sending it to reviewers. A high proportion of publications on investigating plant extracts also have very little value because the results are not reproducible between different laboratories. These aspects motivated the writing of this paper to address problems in the methods and to make some recommendations.

## Methods

### Agar diffusion studies

Before more sophisticated chemical methods were developed, the antimicrobial activity of antibiotics was determined by biological assays. The most popular of the different methods was by using diffusion of an active compound into an agar plate of microorganisms.

In brief, liquid hot agar containing the required growth medium was inoculated with the test organism and then poured into a sterile Petri dish to solidify. A hole was then punched into the agar and filled with the solution containing the antibiotic. Alternatively, a filter paper disk (frequently 6 mm diameter) containing the test solution, was placed on the agar. The Petri dish should then be placed in a refrigerator for the test solution to diffuse into the agar before it is incubated. By the diffusion of active compounds there would be a concentration gradient from the edge of the hole or the paper disk. The microbial growth would then be inhibited by the effective concentration of the antibiotic. The zone of inhibition from the edge of the paper disk or hole is related to the concentration of the antimicrobial compound in the solution.

#### Factors influencing the zone of inhibition

The book Theory and Practice of Microbial Assay [8]) provides an excellent background to agar diffusion methods. Apart from general aspects such as preparing the culture medium, preparation of the inoculum or spore suspension and the importance of making at least six measurements of inhibition zones, they identified the following factors that will influence the results:

- “The concentration of the antibiotic in the test solution.
- The volume of the test solution in the hole or on the paper
- The density of the inoculum.
- The duration and temperature of the diffusion phase before incubation.
- The thickness of the agar medium.
- The composition of the medium
- Incubation temperature”

To achieve comparable results between different laboratories these aspects all have to be addressed. In practise this hardly ever happens when examining the activity of plant extracts, making it practically impossible to compare results between different laboratories.

#### Dose-response relationship

Many scientists accept that there is a linear relationship between antimicrobial activity and the zone of inhibition in agar diffusion studies. In practise over a wide range of doses the square of the zone of inhibition has a linear relationship with the logarithm of the dose [8]). I have not seen that this approach has been used in examining the activity of plant extracts in any papers that I have evaluated or read.

Some authors have tried to address the problem by determining the zone of inhibition of a positive control antibiotic and then calculating the relationship. In papers that I have evaluated they unfortunately, do not use the same concentration of the extracts with the positive control. They have also not calculated the square of the zone of inhibition in assessing activity.

#### The physico-chemical problem associated with agar-dilution studies

Because the agar is an aqueous preparation, non-polar compounds will not diffuse as well as polar compounds. In several cases it has been shown that the intermediate polarity compounds have the highest antimicrobial activity and polar extracts such as water does not extract antimicrobial compounds from many plants [9, 10]. By using bioautography it could be shown that many plant species contain several antimicrobial compounds and they are usually relatively non-polar based on the polarity of the best extractants and Rf values in bioautograms [11].

The agar-diffusion method could be useful with a single compound with a known polarity. Even in such a case if the polarity of the positive control differs much from that of the single compound, comparisons may not be valid. Because plant extracts frequently contain several antimicrobial compounds with different polarities the agar-diffusion method is therefore not useful to determine antimicrobial activity [11].

#### Comparison of MIC and zone of inhibition

There have been different definitions of the minimum inhibitory concentration (MIC) [12]. It is widely accepted that the MIC represents the lowest concentration of a substance that inhibits the growth of a microbe and that this is a better way to express the antibacterial activity of a compound or an extract than the minimum lethal concentration. MIC is also the way in which the activity of antibiotics is presented.

The MIC can also be determined by a revised agar diffusion method if different concentrations of the test solution is added to the hole or filter paper disk. The lowest concentration where no growth inhibition is seen, is registered as the MIC.

Some authors have determined the zone of inhibition as well as the MICs of extracts and compounds by serial dilution assays [13]. I have calculated the correlation coefficient between zone of inhibition and MICs of a methanol extract of *Caesalpinia bonducella* seed, α-amyrin isolated from the seed and the positive control kanamycin against 12 Gram negative bacteria [13]. The correlation coefficients were 0.0078 for the plant extract, 0.2451 for α-amyrin and 0.0009 for kanamycin. It is clear that totally different results are obtained in determining the zone of inhibition by agar diffusion as a parameter of antimicrobial activity and the MIC determined by serial dilution studies.

Agar diffusion studies are therefore not acceptable in studying the activity of plant extracts because the polarity of active compounds greatly affects the results. Furthermore, many aspects have to be addressed to ensure some reproducibility between laboratories and zones of inhibition do not provide useful information about a plant extract or compound.

Some authors have used agar dilution studies where different concentrations of the extract or isolated compound is made in agar and then microorganisms are streaked on top of the plate [14]. This method overcomes the problem of diffusion of the active compound in the agar, but requires large quantities of extracts or isolated compounds and is also much less sensitive than microplate serial dilution methods [14].

In some cases, authors do a preliminary screening by using agar diffusion as a first step and then determining MICs by serial dilution at a later step. There is not much logic in this approach because many positive leads of extracts containing non-polar antimicrobial compounds will be missed.

## Results

### Methods based on serial dilution to determine MIC

There have been several papers discussing methods to be used to determine MICs in blood or body fluids in clinical practise [15]. EUCAST the European Committee on antimicrobial susceptibility testing provides guidelines in the case of clinical work on humans and animals. These guidelines may be obligatory in the European Union. Plant extracts, especially of leaves using an extractant that extracts polar and non-polar compounds, probably contain more diverse compounds than body fluids do and contains compounds that interfere with the EUCAST procedures. Many plant extracts also contain several antimicrobial compounds that differ in polarity. The methods used for clinical studies are therefore not relevant to investigating the antimicrobial activity of plant extracts.

#### Turbidity as indicator of microbial growth

Serial dilution of large volumes of test solutions in test tubes with the subsequent addition of microbial cultures and visual observation of turbidity has been used for many years. This method requires large quantities of the test compound. Furthermore, in my experience, when plant extracts are added to the complex microbial culture media, precipitation may occur complicating the results. This is also the case when serial dilutions are made in microplates and turbidity is measured by a microplate reader. In some cases, the cells clump at the bottom of the microplate [14]. Depending on the extractant used the colour of plant leaf extracts may also make it difficult to quantify the turbidity.

#### Fluorescence as indicator of growth

Chand et al. [18] used a spectrophotometric method based on the presence of esterases in microorganisms. By adding fluorescein diacetate to the culture the esterases in growing microbes would hydrolyse the fluorescein diacetate to yield a fluorescent product. This method has not found wide application and the problem of autofluorescence by bacterial cultures was mentioned by Mann and Markham [12].

#### Using resazurin as a measure of growth

The need for a different method to examine plant extracts was identified and discussed by two papers published in 1988. Mann and Markham [12] worked on essential oils and applied resazurin, a redox indicator that is used in the dairy industry. Microbial growth is indicated by an irreversible change in colour from the blue of resazurin to pink resofurin in the first phase and in the second phase to the colourless dihydroresofurin. Eloff [14] used tetrazolium salts as indicator of microbial growth.

#### Using tetrazolium salts as indicator of growth

Tetrazolium compounds can act as electron receptors in the electron transport chain of microorganisms and in the reduction process be changed from a colourless product to a coloured insoluble formazan. Eloff [14] compared the use of three tetrazolium compounds (2,3,5-triphenyltetrazolium chloride [TTC], tetrazolium red], 3-(4,5-dimethylthiazol-2-yl)-2,5-diphenyltetrazolium bromide [MTT, thiazolyl blue] and p-iodonitrotetrazolium violet [INT]) as bacterial growth indicators. In all cases there was a change of colour within 10 to 60 min. TTC was however, reduced by oxygen in air and changed colour without the presence of a microorganism. MTT and INT worked well at a lower concentration 0.2 mg/ml. The formazan formed with INT was stable but the colours of formazans formed with TTC and MTT faded after some time. Activities obtained by agar diffusion was 3–20 times lower than values found with the INT microplate serial dilution method.

INT also worked as well with several fungi [16, 17]. For mycelial fungi the best approach was to let the fungus grow on agar until sporulation, followed by collecting conidia with a sterile swab, suspending the conidia in growth medium and then treating it as yeasts. To minimize fungal contamination of the laboratory, it is useful to seal the microplates before incubating. It also worked well if INT is added directly after serial dilution.

**Fig. 2:**
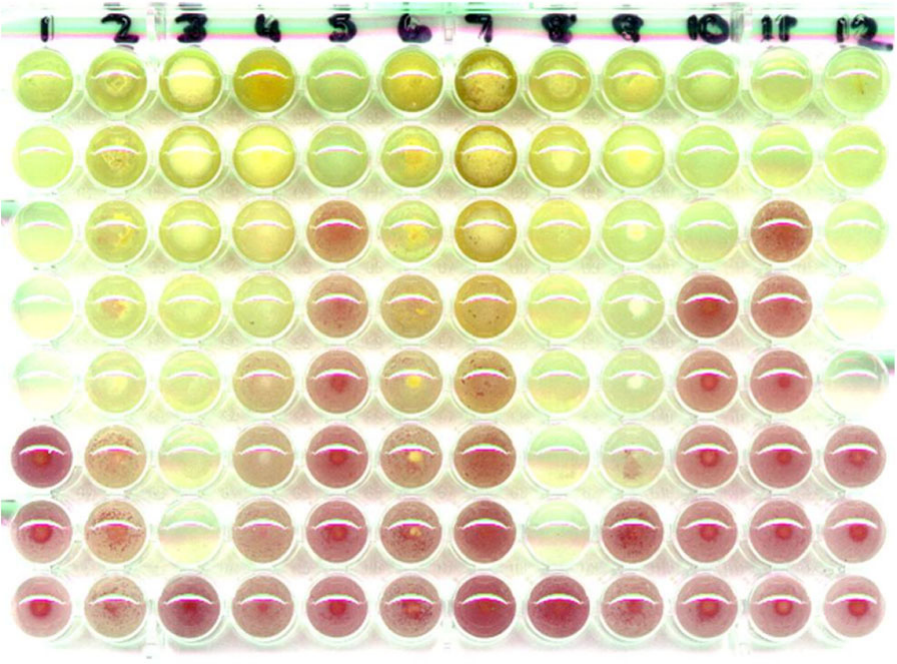
Microplate of serially diluted acetone extracts of 12 *Combretum* species treated with INT and incubated overnight. Microbial growth of *Staphylococcus aureus* indicated by red colour. If started with 10 mg/ml extract the MIC of lane 11 was 1.25 mg/ml and of lane 8 was 0.04 mg/ml

An example of the colour developments in serially diluted plant extracts incubated and treated with INT is provided in Fig. 2.

#### Evaluation of different redox indicators for determining MIC

Klanchnik et al. [19] used a totally different approach by determining the adenosine triphosphate (ATP) activity by bioluminescense as a measure of microbial growth. They also evaluated the use of TTP, INT and resazurin and they found that INT gave the same MIC values as ATP concentrations determined by bioluminescense. Based on the ease of the process and cost they proposed that INT should be used for aerobic bacteria.

In their hands INT did not work well with micro-aerophylic *Campylobacter* isolates. We have however found that INT worked well with micro-aerophilic bacteria involved in tooth decay. The addition of anaerocult A in a sealed container led to good results in bioautography. The procedure using INT also worked well with the anaerobic *Clostridium perfringens* cultured in deoxygenated blood tryptose (BTA) agar incubated in an anaerobic cabinet (6% oxygen; 10% carbon dioxide and 85% nitrogen) [21]. The difference may be related to the inoculum size used in both cases.

Cos et al. [20] stated that where the INT formazan is insoluble, resazurin stays in solution after changing colour. This makes it possible to measure the activity accurately by a microplate reader and to determine EC_50_.

## The organism and inoculum used for assays

### Organisms used

There are large differences in sensitivity between different isolates of the same microbial species. It therefore makes much sense to use standardized isolates. The American Type Culture Collection (ATCC) have a number of isolates that they maintain and provide to scientists. These isolates have been used to compare the activity of other antibiotics by the National Committee for Clinical Laboratory Standards [15]. There is much to be said for using these isolates to enable comparison of results between different laboratories.

### Inoculum size

Microbial infections of animals, usually starts with a low number of organisms present. It is well known that microorganisms initially have a lag phase where there is little or no growth. This is followed by an exponential growth phase and later a stationary growth phase. Bacteriologists have tried to understand the mechanism causing the different growth phases [22]. Some authors state that inoculum size has an influence on the MIC [23]. This is probably true with a low inoculum because cells need time to adapt to the environment. With a very large inoculum this adaptation is minimized. A large inoculum would also minimize the time of cells in the stationary phase to get into the exponential growth phase. When a 1% inoculum of *Staphylococcus aureus* in Müller-Hinton broth was incubated for 1, 3, 6 or 24 h before determining the MIC using a 50% inoculum in the serial microdilution assay, there was no change in the MIC [14]. This obviates the requirement for counting and adjusting cell numbers before determining the MIC. A large inoculum also makes it possible to work under clean but non-under sterile conditions. When the initial 1% inoculated culture incubated overnight was stored in a refrigerator for 1, 10 or 14 days there was no difference in MIC when incubated for up to six hours and stored at c. 5 °C for up to 14 days [14].

## The solvent used for extraction and for dissolving dried extracts in the bioassay

Many different extractants have been used to extract antimicrobial compounds from plants [6]. Based on several different parameters acetone had the highest score of different extractants (acetone, ethanol, methanol, methanol:chloroform:water (12:5:3): (Table 1) [24].

**Table 1:** Comparison of extractants on different parameters based on a five point scale [0–4] and with different weights allocated to the different parameters. [A = results for *Anthocleista grandiflora* and C = results for *Combretum erythrophyllum*]

	weight	Acetone		EtOH		MeOH		MCW		MDC		Water	
parameter		A	C	A	C	A	C	A	C	A	C	A	C
quantity extracted	3	6	3	9	6	12	12	12	12	3	3	9	9
rate of extraction	3	12	15	12	12	12	12	12	12	15	15	9	9
number of compounds extracted	5	20	20	10	15	15	20	10	15	10	15	5	5
number of inhibitors extracted	5	20	20	0	10	20	15	20	20	20	15	0	0
toxicity in bioassay	4	16	16	8	8	0	0	8	8	8	8	16	16
ease of removal	5	20	20	5	5	10	10	10	10	20	20	0	0
hazardous to use	2	8	8	8	8	2	2	6	6	6	6	8	8
total		102	102	52	64	71	71	78	83	79	79	47	47

Reprinted from [24] With permission of Journal of Ethnopharmacology

With the two plant species examined acetone gave the best results by far. This was confirmed by many studies on extracts of several other species [8, 25, 26].

## Positive and negative control treatments

To ensure that the method has worked well and to compare the activity of the extract or isolated compound a positive control of a standard antibiotic should be included. The positive control should be tested at the same concentration as the plant extract.

A negative control of the solvent (or carrier) used to dissolve the extract or isolated compound should be included to ensure that any activity on the microorganisms is not caused by the solvent. It would be useful if the solvent could extract polar as well as non-polar compounds and be miscible with water. To determine the activity of volatile oils there are many problems if the solvent is not miscible in the aqueous microbial growth medium [19].

The average MIC for different extractants that are miscible with water against fungi are DMSO 45%, acetone 51%, ethanol 30% and methanol 32% [27]. The highest concentration of the solvent that microorganisms are subjected to in the INT serial dilution method [14] is 25% because the extract is diluted 1:1 in the first well and another 1:1 dilution by the addition of the 50% microbial inoculum. Because acetone is also a good extractant for essential oils and the sample can easily be recovered by evaporating the acetone, we use acetone as solvent. In several thousand assays we have never found any growth inhibition by the 25% acetone present in the first well.

In rare cases we have found that a dried acetone extract did not dissolve in acetone after drying. In such a case it helps to determine the concentration of an aliquot of the extract, to calculate the concentration of the extract followed by either evaporating some acetone or adding acetone to attain the required concentration [28].

## Selection of plants to investigate

Many researchers select the plants they work on based on traditional use against infections. Because traditional healers in general only have water available as an extractant and most antimicrobial compounds in plants have intermediate or non-polar characteristics [8, 10], in my experience this is not a good basis to select plants in order to search for new antibiotics.

This does not mean that water extracts or traditionally used medicinal plants do not work. Because many aqueous plant extracts contain anti-oxidant compounds, the efficacy of plant extracts used traditionally may not be based on inhibiting microbial growth per se, but rather on stimulating the immune system of the patient. The extracts of plants containing tannins or saponins that are soluble in water may have antimicrobial activity. In comparing plants used traditionally with plants randomly selected we found no statistically significant difference in antimicrobial activity (unpublished results).

When the antimicrobial activity of acetone leaf extracts of 537 tree species was determined against seven important bacterial and fungal pathogens, there was a statistically significant difference in the antimicrobial activity between orders of trees [17]. The probability of finding extracts with good activity increased threefold and fivefold for Gram-positive and Gram-negative bacteria respectively between different tree orders. Focused collection based on plant taxonomy may therefore be feasible to get a higher hit rate.

## Additional aspects

A statement such as “this plant has antimicrobial activity” is totally useless unless the dose is specified. All plants have antimicrobial activity if the dose is high enough. There were practically no acetone leaf extracts with MICs higher than 2.5 mg/ml after 714 acetone leaf extracts of 537 tree species were examined [17]. Many authors consider that only extracts with MICs lower than 0.1 mg/ml are interesting in a search for potential new antibiotics. There is therefore not much chance of getting a paper published with MICs higher than 0.5–1 mg/ml in good journals.

## Evaluation of success using serial dilution with INT

The original paper describing the use of INT to determine the MIC of plant extracts [14] have been cited more than 1830 times in Google Scholar. Many other authors have also used it, but did not cite the original publication. In an analysis of different techniques Klancnick and her colleagues [19] recommended using the INT based serial dilution technique instead of resazurin because it provided similar results as measuring ATP content.

By using this method on acetone leaf extracts of 537 southern Africa trees, leaf extracts an average of 4.6% of the species investigated had an MIC of 0.04 or lower (unpublished results). Many publications have emanated from an in depth investigation of extracts of species with excellent activity. Some of the results have led to potential commercial application in plant production [10], animal production [29] and human health [30].

This method also led to the discovery a potential new antifungal framework molecule with higher antifungal activity than current antifungal agents and very high animal safety [31].

In the Phytomedicine Programme at the University of Pretoria we have found that resazurin works better than INT for slow growing mycobacteria. This has been confirmed by other authors working on mycobacteria [32].

## Discussion

Agar diffusion methods are not acceptable and cannot be used to determine MICs of plant extracts due to the insensitivity, lack of diffusion of non-polar molecules into the aqueous agar matrix and difficulty of obtaining results that are reproducible between different laboratories. There has been a move away from using agar diffusion methods to determine the antimicrobial activity of plant extracts with the aim of discovering new antibiotics. Between 1997 and 2008, 25% of publications in South Africa used only disk diffusion methods to determine antimicrobial activity of extracts (van Vuuren, [33]).

Large scale serial dilution methods and measuring turbidity may be acceptable if there is no precipitation occurring after the plant extract is added to the complex microbial growth medium. An advantage of this method is that it is easy to determine the microbial growth curve.

Serial microplate dilution methods using INT or rezurasin as indicators of growth works well, provide reproducible results for MICs and could also provide information on minimum lethal concentrations if cultures are grown for longer time. It has been shown that MICs determined by using serial microdilution with INT as indicator of growth are realistic in subsequently growth studies of colonies plated on agar of cultures grown at MIC, 0.5 X MIC and 0.25 X MIC concentrations [34].

## Abbreviations

ATP: Adenosinetriphosphate;
INT *p-*: Iodonitrotetrazolium violet
MIC: Minimum inhibitory concentration

## Authors’ information

The author is the founder of the Phytomedicine Programme in the Faculty of Veterinary Science at the University of Pretoria and c. 50 MSc and 50 PhD students graduated under his supervision leading to > 315 publications in peer reviewed scientific journals. He is a member of several Editorial Boards where he had to evaluate many manuscripts in this field.
